# Meeting of the Buffalo Medical Club

**Published:** 1882-09

**Authors:** 


### Society Reports.

### Meeting of the Buffalo Medical Club.

The regular semi-monthly meeting of the Buffalo Medical Club, was held Aug. 9th. The subject for discussion was the Revised Code of Ethics of the New York Medical Society. Dr. H. R. Hopkins presented the affirmative argument, and Dr. W. S. Tremaine, the negative. We present their papers in full.

### DR. HOPKINS' PAPER.

Mr. President and Fellows—In presenting an affirmative argument upon the question of the wisdom of the Revised Code of the New York Medical Society, I am not unmindful that such an argument is not in harmony with the sentiments of a large majority of our profession. My excuse for differing with that majority is the simple fact that they have not yet deigned to set forth any facts or arguments to show why the new code is not a wise rule of conduct. Of resolutions, declamations and proclamations there has been more than sufficient; of pertinent facts, argument and reasoning, we have had a continuous dearth. I state this before hearing the able argument of our Fellow who is to present the negative side of this question, in order that, in case it becomes my duty to report my conversion, he may have the greater glory.

Our Code of Ethics is a formal expression of the sense of the medical profession, of the ideal relations which should exist between the individual members of the profession, between the members of the profession and their patients, and between the medical profession and the public at large. The expounding of such a code is a solemn and pre-eminently important act, as it attempts to fix the rights of the individuals of the profession, the rights of the sick and afflicted who may seek our aid, and the rights of the individuals of our people at large, so far as those rights are concerned with our duties or privileges.

It must be plain to all that a code of ethics can not be properly conceived which asserts the rights of one portion of our people to the exclusion of the rights of another, no matter however few in number or insignificant in influence that minority may be. A code of ethics may ask the individual of its class to deny himself, and to forego some temporary advantage for the sake of the general good, but it can never ask an individual to wrong, or to debar the rights of another upon any plea whatsoever. The associated profession is an important member of that family of associations, which make the state, and her official formulas must be written in the key of universal tolerance, in harmony with the principles for, and by which, the state was founded.

It is frequently remarked that this is a trivial matter which concerns the profession only, but to correct so false an impression it only needs to be stated, that in fixing our relations to our patients, we in so far define and limit the rights of those patients, and in nominating the relations which the profession should maintain to the public, we, in the same degree, limit the rights of the public. That this is the correct view of the importance of our code, there can be no doubt; consequently no more momentous matter can come to our consideration.

We saw that our code of ethics must be so formed as to zealously guard the rights of the people, not of our class, for whom we speak. I am glad of the chance of saying to you, that the codes of our fathers, in this respect, could defy criticism. Before proceeding, however, to this part of our argument, we will do well for a few moments to consider the question, why we have a code of ethics:

The medical profession of this State, as an organized factor of society, dates from April 4th, 1806. On that day the people recognized the individual physicians of the State, and raised them to a privileged class distinguished from their fellow citizens, by the possession of certain rights and priviliges, duties and responsibilities. Chief among these rights was that of assembling and organizing State and County Medical Societies, into whose hands was placed the entire responsibility of the profession of the future. This control of the future of our profession was provided for by a grant of authority to our State and County Medical Societies, to enact by-laws for the government of their members, relating to the granting of licenses to practice, and to the conditions upon which members were in the future to be admitted to such societies.

The New York Medical Society, incorporated April 4th, 1806, was duly organized on the first Tuesday in February, 1807, and at that time adopted by-laws containing the following ethical provisions: "Any member of the said society who may have "been convicted of any serious offense against the laws of the "State or of the United States, or who may guilty of gross im-"morality, or who shall have improper pretentions to any "specific or nostrum, or who shall be repeatedly be guilty of im-"proper conduct in the duties of his profession, or his behavior "in this society, may be expelled at an anniversary meeting "upon a vote of two-thirds of the members present." This with a by-law declaring that it is inconsistent with the dignity of the medical profession for physicians and surgeons in their corporate capacity to arrange and fix professional charges was the wisdom of the fathers upon the subject of ethics, and by these principles they were governed for the years between 1807 and 1823. At this time a formal code was adopted entitled a "System of Medical Ethics." This system was the result of the labors of a committee of the most eminent medical men of their times. The committee was appointed February 8th, 1821, was ordered to sit again February 5th, 1822, and made their report February 6th, 1823. This code was in force from 1823 until 1849, when it was superseded by the code of the American Medical Association, which our State Society at its last meeting, February 7th, 1882, revised.

I would call your attention to one provision of our code of 1823, as illustrative of the temper of the medical profession ot that time, Section VIII, Specifications of Medical Ethics in Practice: "Honor and justice particularly forbid a medical prac-
"titioner infringing upon the rights and privileges of another
"who is legally accredited, and whose character is not impeached
"by public opinion or civil or medical authority, whether he be
"a native or a stranger settled in the country. There is no
"difference between physicians but such as result from their
"personal talents, medical acquirements, or their experience, and
"the public from the services they receive, are the natural judges
"of these intellectual advantages."

You will observe that the expounders of our primitive codes were keenly alive, to the importance of recognizing and protecting the rights of the people for whom they assumed to speak. You will also remark the close similarity of language between the codes of 1823 and 1882.

"Legally accredited" says 1823.

"Legally qualified" responds 1882.

I must hasten to briefly sketch the situation which was presented to the committee appointed at the meeting of the New York Medical Society in 1881 for the purpose of revising our code. They find in operation a code of alleged ethics which practically forbids the members of the medical profession from recognizing as physicians the members of the homœopathic profession. This code has been in operation since 1849, and since 1857 its operation has been as follows: An attempt to abridge the rights of the homœopathists by refusing to recognize them as physicians; an attempt to abridge the rights of the individual members of that profession by not meeting them in consultation, and an attempt to abridge the rights of our people who wish to employ homœopathists and, when desired, to have the benefit of our consulting attendance. This construction of the code of 1849 is based upon the action of the law of 1857, creating the homœopathic profession and the assumption that a physician is not warranted in refusing to recognize, as a physician, another of "good moral character and legally authorized to practice medicine," because he does not like the manner in which he parts his hair.

That to be entitled to all the rights and privileges of the practice of physic and surgery under the laws of the State of New York, is to be entitled to the services of other physicians similarly authorized, in consultation, when desired, by himself or his patients.

Besides these wrongs in our modern ethics, there was the greater wrong to ourselves; to our record as scientific students, in the attempt to maintain the untenable, unscientific negation. That a well educated, honest and careful homœopathist in a life-long study of disease, can not observe anything which can be an addition to the knowledge of a member of our profession. In my judgment the existence of these wrongs in our code of alleged ethics was sufficient reason for a revision.

Practically the most dangerous wrong in our code is the wrong we do the people who patronize homœopaths. It had been the habit for members of the medical profession to fight homœopathy and occasionally to fight homœopathists, and it was our right and duty so to do any time prior to the year 1857. But at that time, in spite of all our fighting, the homœopathists won from the people of the State of New York recognition as physicians. The birthright which was bestowed upon our profession in 1806 was not taken from us, but was extended to them, and the medical future of the Empire State became our joint care. Unfortunately the habit of fighting homœopathy had become so fixed that we have continued the practice up to the present, in forgetfulness that now we are not fighting the members of the homœopathic profession, but that we are attempting to fight the people of the State of New York.

The statutes have declared and the courts construed the statutes to mean, that they are physicians, while we continue to shriek in hysterical tones: "But we tell you they are quacks." We may be positive that this course is not right, and absolutely certain that it is not expedient. This matter must be so stated that it cannot be misunderstood and at the risk of being tedious. I will repeat what seems to me to be the basis of my argument.

The rights of the New York medical profession are legislative grants from the people. We exist by the will of the people, for the good of the people, and the people have the right to ask, and if need be, to demand our services. The claim of the people upon the services of our profession lies upon the same plane as the right to life, liberty and the pursuit of happiness, and it does not rest with us to say for what cause, or when, the people have forfeited that claim. Every right, which we enjoy, has also been possessed by the homœopathists since 1857. Our code of 1849 fails to recognize the rights of the homœopathists as a profession, as individuals, and the rights of the people who wish their services.

Since the year 1857 our code of ethics has been at variance with and in defiance of the laws of our State; has been sectarian, intolerant, and unscientific, and its reformation is called for by every argument of fidelity, justice and expediency.

As a profession we seem to have very misty ideas as to the origin and nature of our rights. I have conversed with some of our leading men who seem to think that we have an exceedingly ancient, or, perhaps, divine commission; that, by virtue of our Hippocratic oath, we are responsible only to the traditions of the profession, frequently another name for party prejudices, whereas the fact is simply this: That our profession owes its existence, and we owe our individual professional rights to the people, and as our rights and privileges constitute an exceedingly valuable franchise, the law zealously guards that franchise from spoliation.

This we seem to be able to comprehend as applied to ourselves, but utterly unable to grasp or to apply to the homœopathists, although we were fashioned by the same hand, with equal rights and responsibilities. Unless these propositions and this reasoning can be overthrown, the need of revision of our code is demonstrated.

Do not let anyone understand me as maintaining that the action of the Legislature creating the homœopathic profession was in any manner or degree wise, for such is not my mind, it is simply my opinion that it does not rest with us to pronouuce upon the wisdom or the unwisdom of that legislative act. We had, and still have all the responsibilities we can well account for, and the people reserve to themselves the right of sitting in judgment upon the consequences of their enactments. The code of 1823 says: "*The public from the services they receive,* "*are the natural judges of the respective advantages of physicians.*" Neither is it for us to consider in this connection the scientific aspect of the homœopathic faith or the advantages, or otherwise of the homœopathic practice. It would seem that in its corporate capacity the medical profession of this State has now no call to consider or discuss either part of this question. The people have desired that the question of homœopathy shall be put to the experimentum crucis of actual practice and open observation. The medical profession had the opportunity, and the code of 1882 gives it to us again, to see that the conditions of the experiment are accurately and faithfully observed, but we have no call to either plead the case or boycott the jury.

Again it seems that there can be no question of the incorrectness of the code of the American Medical Association, viewed from the scientific standpoint. As before stated, the logical action of the code was this: No matter how great a man's intellectual qualifications may be, or how rich and varied his observations, if he is a homœopathist, he has never seen anything, he can never see anything which can inform us or benefit our patients.

This is the action of the code of the American Medical Associatidn, and for revising this in our State so as to allow our members to act upon their own judgments when asked to meet legally qualified physicians, that Association practically closed its doors upon the profession of the Empire State.

The absurdity of this action gets to be pretty plain when we look at the history of the profession in our own State. In 1806

the people put the future of medical profession into the hands of the corporate bodies, the State and County Medical Societies. By their by-laws they were to say who should study medicine; how they should study; how long they should study; when and how they might graduate; how they might join their societies and become practicing physicians and surgeons, and how they should continue in, or be expelled from that practice; never was a more royal trust more freely bestowed.

And what did we do with it? We turned to and filled our state with private corporations whose business was to sell the degree M. D., the same being a license to practice physic and surgery anywhere in this State or in the United States.

What preliminary education did we require of these candidates for the honor and responsibilities of the practice of medicine? None at all.

What term of study did we exact of them? Not any. How many courses of lectures must they attend? Not one. What clinical advantages must he have? He was never inside of a hospital, save as a patient. What examination to test his ability, to meet the duties for which he is about to be certified as well as qualified to perform, must he pass? None at all. If he was capable we were glad of it and hoped he would stay in the city and send us students. If he was incompetent, we were sorry; but if we did not graduate him, our neighbor in the next town would and we needed his money.

What was the result? The people, the natural judges of the respective advantages of different physicians, said in 1857: We have found the homœopathists to be about as good as our regular doctors, at any rate they are not much worse, and we will see how they will get on together.

How does this action affect us? We, at once, are all alive to the great danger which the public are bringing upon themselves, and we warn them in our code of ethics to beware of the homœopathists, who are not physicians at all. So great is our solicitude that we will not permit our illiterate, incompetent holder of a purchased diploma, to take for himself or for his unfortunate patients, the council of a man, who has all the advantages of literary, scientific and professional training, which the universities and hospitals of Europe and America can furnish, but who is a homœopathist. The fact that his opinion differs from ours as to the curative action of drugs, makes him dangerous in spite of his exalted personal character and his extended scientific and professional acquirements.

The American Medical Association disfranchises one-tenth of the medical profession of the United States for recognizing this man as a physician, but is as dumb as an oyster at the practice of wholesaling medical diplomas.

You may look far and wide before you can match the absurdity of the spectacle of the American Medical Association, straining at the gnat of a homœopathic globule, after having annually for 35 years swallowed, with alleged satisfaction, the camel of American medical education.

Before concluding I must remark in passing upon what seems to be a unanimous misconception of the supposed action of the code of 1882.

It would seem from the reports of all the official actions and most of the individual utterances upon this subject, from the august American Medical Association down to the humblest local medical society, that the logical action of the code of 1882 is to recognize homœopathy, hahnemanism, infinitesimalism, and all the alied delusions.

That to meet a man in consultation, is to extend to him full fraternal recognition, to undertake to confirm his diagnosis and to support his treatment. This seems to be the popular conception of the action, and the only possible action of our new code; and I wish simply to remark that nothing could be more false or misrepresenting. To my mind the action of the new code is: first, to say to the people we bow in acquiescence to your authority and recognize the homœopathists as physicians; second, to say to the homœopatists, " we will meet you where, and when you will, and treat you with exact fairness and justice, recognizing that consultation is for the benefit of the patient, and that in case we disagree, as we are likely to, regarding diagnosis and prognosis, and are sure too about treatment, we will be governed by our code for such cases made and provided." That legal recognition and consultation, means or necessitates full fraternal recognition and relationship, I am sure never for a moment entered the minds of the framers or advocates of our new code. The general impression seems to be that, willingness to consult implies on our part all the rest; to my mind it simply implies formal official recognition of the relations of the homœopathists to the State. We accept their credentials and allow the developments of the future to decide whether their relations to us will ever be more than formal recognition. The laws of the state, the temper of our people, and the genius of our institutions demand this, but as we are not allowed to proscribe them for opinion's sake, they can not coerce our endorsement of their doctrines.

In conclusion I would call your attention to a few reasons in support of the expediency of the action of the New York Society in adopting the code of 1882. By expedient I do not mean the lower as preferred to the higher interest, the present to the future good. In my conception expediency has nothing in common with worldly wisdom, cuteness or sharp practice. I use the term only in its higher and more comprehensive meaning as synonymous with fit, appropriate, judicious, wise.

For instance, one might say that it is expedient for us everywhere and always to keep in mind that we, as members of a learned and honorable profession, stand among the higher orders of society, and that our lives and characters should be in harmony with the dignity and responsibility of our position. Also, that it is expedient that we should not forget that medicine is neither an art nor an exact science; that the problems we have mastered, are to those which lie before us, as the drop to the bucket; that we have small occasion for sweeping generalization, save in mentioning what we do not know; that from the nature of our work, from the immensity of the responsibility involved in our work—the preservation and restoration of health—we can not afford to shut our eyes to the most seemingly trivial fact, connected with the history or the management of disease. Who would dare belittle the importance it would be to medicine to have the chance of correcting our views of the life history of disease, by the opportunities which homœopathy continually *presents us of studying disease uninfluenced by treatment.* When a reasonable number of our people are daily offering themselves as material for this experiment, it is certainly expedient for the medical profession to be critical students and observers of the process and its results.

Notwithstanding the rapid strides of histology, pathology, physiology and chemico-physiological therapeutics, medicine will for a long time remain as it has ever been in the main, a matter of empirical observation, and it will be expedient for us to first demonstrate to the satisfaction of the people, that homœopathy has not a single kernel of wheat to the bushel of chaff, before issuing our decree of proscription

There is truth in homœopathy, or there is not; if there is, it belongs to us, and it is expedient for us to determine this fact, and to take our own. If the truth is not there, not even the bad legislation of its enemies can keep it alive.

In conclusion it is expedient for us as individuals to live as humble students of the yet-to-be science of medicine, and as obedient citizens of the State, remembering "that the submission of a free people to the representative authority of government, is no more than a compliance with laws which they themselves have enacted," remembering, that if we have put upon us burdens grievous to be borne, the patient bearing of them will hasten the time of our deliverance. It is expedient for the medical profession to regulate her official formulas in harmony not only with the letter of the law, but more than this, in harmony with the spirit of the times, which writes experiment and tolerance, in place of dogmatic assertion. It is also expedient that we discuss this question without class bias or prejudice, which makes me think, that the full measure of expediency is not always easy of realization

---

### DR. TREMAINE'S REPLY.

MR. PRESIDENT AND FELLOWS—In obedience to your vote, I shall endeavor to reply to the arguments of my Fellow on the affirmative side of the question, Shall the action of the New York Medical Society in its revision of its code in regard to consultation be sustained?

I confess, sir, it is difficult for me to approach this subject with anything like an ordinary degree of patience. Why should a body of intelligent, well educated men find it necessary to discuss such a question? In looking for an answer, I discover in this good city of Buffalo, that, notwithstanding its large body of accomplished and able medical men, it is positively reeking with the poison of medical heresies. "Pathies" and "isms" stalk abroad at noonday and find their support and countenance from a large class of the community who would probably act differently were they well instructed in regard to the narrow-minded bigotry and pretentious humbug of all "isms" and "pathies," no matter what prefix soever may be used to the particular pathy. I pass over the ill-logical demand of my learned friend who calls upon the majority "to prove a negative" and asks your attention for a moment to exactly what is said in the new code under the head of "Rules Governing Consultations:" "Members of the Medical Society of the State of New York and of the medical societies in affiliation therewith, may meet, in consultation, *legally* qualified practitioners of medicine. [The italics are mine.] Emergencies may occur in which all restrictions should in the judgment of the practitioner yield to the demands of humanity."

On the face of it this gracious permission seems especially fair and broad-minded, and one wonders why such permission should be necessary.

Certainly no such liberty is required as regards *qualified* practitioners of medicine. The force then of this lies in the estimation accorded to the word "*legally*."

I am not familiar with the exact terms of the statute which *legalizes* the practices of medicine in this State, but fortunately my learned Fellow has furnished me with the information with which to attempt his "conversion."

The true inwardness of this section of the code of ethics my friend supplies when he uses the term "members of the homœopathic profession." ! ! ! Homœopathic profession! I cannot believe that he, an accomplished, educated physician is one of those mistaken mortals who recognize "schools" in medicine, but he has used the term and seems desirious for permission to recognize and consult with what he terms the "homœopathic profession," and tells us that we abridge the *RIGHTS ! !* of the homœopathists by "refusing to recognize them as physicians, and by not meeting them in consultation."

This then, sir, divested of all rhetoric and sophistry, is the especial purpose for which this revision of the code was enacted, and here let one pause to thank our Fellow for furnishing us a true explanation of this apparently harmless and liberal section of the new code.

Be it so! We accept his assertion as the true intention, and this leads us to inquire into what is this homœopathy, and who and what are these homœopathists whom he is anxious to reach out to, and take into fellowship *unasked* and *unsought* by them. I take it, sir, it is fair to judge of any sect by their published creed, and here is the creed of the homœopath.

1st. That diseases are dynamic changes in a vital principle, or in other words, are spiritual entities not depending upon material substance.

2d. That the cure of disease is most easily and completely effected by drugs, therapeutic action of which closely simulates the symptoms of the disease itself, and that the probability of a favorable result increases with the exactness of resemblance or similarity.

3d. That the power of drugs increases with their attenuation, which may be carried to an almost unlimited extent.

4th. That trituration or agitation confers new and augmented power upon the drug so treated.

As to the first of these propositions, I commend to its author the advice of Herbert Spencer, to "translate our words into thought."

I frankly confess my own inability to comprehend its meaning, nor do I believe it possible for any human being to understand what is meant by it.

In regard to the second article of faith, it is in short the new familiar fallacy of "similia, similibus curantur," which I am told is practically abandoned by the disciples of Hahneman.

I learn that the attenuation doctrine is now somewhat limited and does not usually go beyond the "30 dilution." That the higher dilutions, i. e., from the 100th to the 200th are not generally adhered to. In order that my friend may be posted before his consultation, may we not inquire what this 30th or comparatively low dilution means? It means, roughly speaking, drop of the "mother tincture" diluted with a quantity of water equal to more than 40 worlds of the size of ours. But perhaps the consultant avows himself a believer in the 40 dilution, which needs 450 billions of worlds of water, and yet modern homœopathy tells us that people are constantly cured by doses of the 100 dilution.

These dilutions must also be carefully made according to the "Pharmacopœia, Homœopathica, Polyglotta" which directs it to be shaken with ten vigorous downward strokes of the arm. Hahneman, it seems, at first applied only "two shakes of the arm," but later he gives "ten shakes of the arm" as the normal number, and retracts his former direction. Will my friend kindly inform us what "rights of the public" can be secured by consultation with one laboring under this phase of lunacy? Would it not be better that their "rights" be for the time ignored, and their lives and health better protected by our refusal to recognize as physicians persons evidently so intellectually weak as to believe in such wretched nonsense. I scarcely think he or any other member will avail himself of the gracious permission of this wonderful code, even to secure their rights to the dear public. But, sir, it would be an insult to the intelligence of this body to offer at the present time such a work of super-erogation as a refutation of homœopathy.

You, my Fellows, *know* there is no such thing. Assuming then our duty to guard the "rights of the public," so emphatically insisted upon by our Fellow, we should refuse to countenance, directly or indirectly, this delusion which exists only in the imagination of the *soi-disant* educated portion of the community, but rather regardless of "expediency," demonstrated by our action to these deluded people that this is a false and exploded doctrine, no longer adhered to by its self-styled disciples. My friend informs us that there exists in the community a class of legally qualified practitioners, called homœopathists, with whom it is our duty to consult. If these people believe in their published creed, about as much benefit would result to the patient from a consultation with one of them as would in a law suit from a conference between an eminent lawyer of the Buffalo bar and a legal light of the Fiji Islands.

If they do not believe in and practice their theory, but profess to, they are imposters and unworthy of the recognition of honorable medical gentlemen, either professionally or socially. But my friend continues his specious argument and tells us "no matter how great a man's intellectual qualifications may be, or how rich and varied his observations, if he be a homœopath, he has never seen anything, he can never see anything, which can improve us or benefit our patients." I am at a loss, sir, to know what code of ethics makes any such assumption or prohibition. Paradoxical as it appears to me, if it be as my friend implies that there exists an intellectual person with "rich and varied observation," and yet a homœopathist. I know of no law or code which prevents his deriving all the information or instructions that he is capable of at their hands.

I confess I do not see the benefit to accrue to a patient by his pursuing his studies at the bedside of the unfortunate, with his intellectual instructor in consultation. If he desires to dip into the rich mines of knowledge which he seems to think may be locked somewhere in the shallow depths of homœopathy, its publications are open to him, a privilege which he no doubt, in his broad liberality, would accord to the homœopathists, and which, sir, you and, I have no doubt, they avail themselves of liberally without permission.

Possibly there may be homœopathists possessing all the advantages of "literary, scientific and professional training which the universities and hospitals of Europe and America can furnish." I have, unfortunately, to confess my unfamiliarity with such a class, if such there be, and I cannot, of course, deny the assertion. I see no good reason why they should not join in with and add to the great body of regularly educated and professionally trained practitioners of medicine, whether of the "hospitals of Europe or America." *If they are scientific men, earnest seekers after truth, they would naturally associate with others of like character, without any test of exclusive dogma, or especial article of faith, but in the broad, catholic spirit of science*, left free to indulge in any mental gymnastics they please about the action of medicines so long as they do not attempt to carry them into practice in violation of known physical laws.

The American Medical Code of Ethics does not mention directly or indirectly any "pathy," but simply states "a regular medical education furnishes the only presumptive evidence of professional abilities and acquirements, and ought to be the only acknowledged right of an individual to the exercise and honors of

6

his profession. Nevertheless, as in consultation, the good of the patient is the sole object in view, and this often depends on personal confidence. No intelligent, regular practitioner who has a license to practice from some medical board of known and acknowledged respectability, recognized by this association, and who is in good moral and professional standing in the place in which he resides, should be fastidiously excluded from fellowship, or his aid refused in consultation when it is requested by the patient. But no one can be considered as a regular practitioner or a fit associate in consultation where practice is based on an *exclusive dogma*, to the rejection of the accumulated experience of the profession and the aids actually furnished by anatomy, physiology, pathology and organic chemistry." Again, paragraph 6, "In consultation theoretical discussion should be avoided as occasioning perplexity and loss of time, for there may be much diversity of opinion concerning speculative points, with perfect agreement in those modes of practice which are founded not on hypothesis but on experience and observation." Can anything be broader or more liberal? Its requirements are simply *ability* and *respectability*. Surely our friend will not ask us to abolish these tests in order that he may consult with his homœopathic friend! Nowhere in any code of ethics of the medical profession is homœopathy or any other "pathy" alluded to. The plain requirements being a medical education, and a renunciation of bigotry, or in other words "exclusive dogma," but not to weary you, my Fellows, our friend begs the question. The summing up of the whole matter is that he requests us as regularly educated, qualified physicians to condescend to consult with and recognize narrow-minded and bigoted professed practitioners of a popular medical delusion. As well might we ask the great body of learned scientists to step down, and acknowledge the traveling conjuror as one of themselves, because some of his tricks may be explained on scientific principles. My friend has much to say about the law compelling us to recognize the disciples of exclusive dogma, which he terms homœopaths. If he will permit me, the law has no authority whatever in this matter. It is not within the province of the law to dictate a man's professional, literary or social companions. The qualifications they should possess is indicated by the code of ethics as quoted; the prima facia evidence of fitness being the possession of a diploma from a medical college where the sciences, which are essential to a liberal medical education, are fully taught by a competent corps of instructors. This at least is presumptive evidence that its possessor has had the *opportunity* to acquire the necessary qualification. That this evidence is not always conclusive, I deplore as much as any one can, but with the present indifference or ignorance of our Legislatures it is probably the best obtainable. Medical societies and State examining boards frequently reject diplomas from colleges, in their opinion lacking the necessary curriculum, no matter how *regular* they may claim to be. I have no doubt that if my friend knows of instances of diplomas having been improperly given, that such an impropriety would be severely dealt with by the profession, and the unworthy possessor excluded, if the matter was investigated. As a would-be guardian of the *rights* of the people his duty is clear in the premises.

It is puzzling to understand why this special form of delusion, i. e., homœopathy is selected for recognition. Why not in this solicitude for the "rights" of the people include in the prospective benefits from the new code all the other "pathists," such as vita-pathists, hydropathists, mesmerists, magnetic healers and manufacturers of "medical discoveries," etc.?

Are their claims to be neglected and their "rights" ignored, because they are in the minority? I would suggest, instead of wasting time in fruitless discussion about codes of ethics, we all, regardless of our pet theories, unite in an earnest practical effort to furnish the people with properly educated, competent practitioners of medicine, which might be done by having a State Board composed of medical men of well-known ability, and one or more highly educated scientific laymen, which board only should have power to grant a license to practice medicine; that this Board, before so doing, should examine thoroughly into the applicant's proficiency in general educational branches, and professionally upon anatomy, physiology, pathology, chemistry, surgery, obstetrics and hygiene. No member of this board to be connected with any medical college. If an applicant be found qualified by such an examination he will not be likely to make serious mistakes, no matter what peculiar theories in regard to the action of remedial agents he may hold. By adopting this plan we would probably do away with any excuse for "schools'. or "pathies," and so protect the "rights" of the people against ignorance and medical delusion.

